# Artificial Intelligence in Coronary CT Angiography: Current Status and Future Prospects

**DOI:** 10.3389/fcvm.2022.896366

**Published:** 2022-06-17

**Authors:** Jiahui Liao, Lanfang Huang, Meizi Qu, Binghui Chen, Guojie Wang

**Affiliations:** ^1^Department of Radiology, Fifth Affiliated Hospital of Sun Yat-sen University, Zhuhai, China; ^2^School of Biomedical Engineering, Guangzhou Xinhua University, Guangzhou, China

**Keywords:** coronary heart disease, artificial intelligence, coronary CT angiography, deep learning, machine learning

## Abstract

Coronary heart disease (CHD) is the leading cause of mortality in the world. Early detection and treatment of CHD are crucial. Currently, coronary CT angiography (CCTA) has been the prior choice for CHD screening and diagnosis, but it cannot meet the clinical needs in terms of examination quality, the accuracy of reporting, and the accuracy of prognosis analysis. In recent years, artificial intelligence (AI) has developed rapidly in the field of medicine; it played a key role in auxiliary diagnosis, disease mechanism analysis, and prognosis assessment, including a series of studies related to CHD. In this article, the application and research status of AI in CCTA were summarized and the prospects of this field were also described.

## Introduction

Coronary heart disease (CHD) has been a disease with the highest mortality worldwide (1), and early detection and treatment will be beneficial to controlling risk factors and reducing cardiovascular events (2). Currently, digital subtraction angiography (DSA) is the gold standard for diagnosing coronary artery disease (CAD) (3, 4). However, DSA is an invasive examination and possesses some defects, such as it can only display the shape of blood vessels but cannot analyze the composition and nature of the plaques and the cost of DSA is high (2). Coronary CT angiography (CCTA) could use prospective or retrospective ECG gating to collect the optimal phase to reconstruct the images at any heart rate, displaying the main branches of the coronary artery in multiple directions and analyzing the diseased vessels (5). Moreover, CCTA could also provide the basis for cardiovascular risk stratification and treatment decision-making and can be used to predict the occurrence of cardiac events (6). The advantages of CCTA include noninvasiveness, convenient examination, fast speed, and relatively low price, which make it the best choice for clinical screening of CHD (7). In recent years, the number of CCTA examinations has increased year by year and the cardiovascular imaging data have increased rapidly (8, 9). Moreover, because of the shortage of imaging diagnostic talents, the quality of CCTA examinations has declined, diagnostic reports have been delayed, and missed or misdiagnosed cases have also increased. As an emerging frontier technology, artificial intelligence (AI) has been developing rapidly in the medical field, including the field of cardiovascular CT imaging (10–12). In this article, the application and research status of AI in CCTA, including imaging technology and assisted diagnosis, and its future development, are reviewed. For this purpose, studies were searched mainly in the PubMed database, by using “Artificial intelligence” or “Machine learning” and “Coronary CT angiography” as keywords and cross-searched in citations; articles published in the last 6 years were retrieved.

## Artificial Intelligence Technology

**Figure 1:**
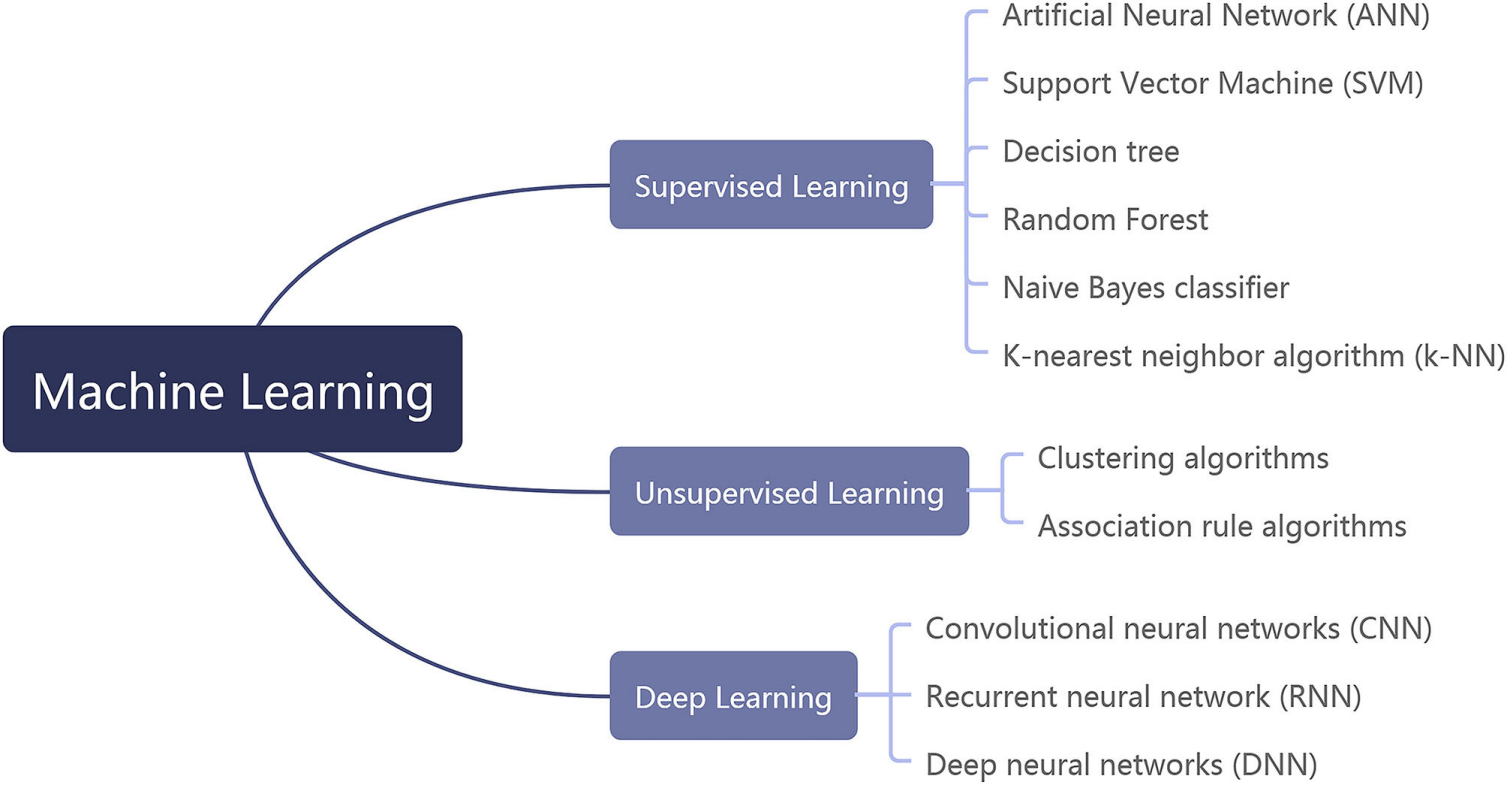
The classification of machine learning.

Artificial intelligence technology can be mainly divided into machine learning (ML) and intelligent computing. ML is the main technology of AI, which includes supervised learning, unsupervised learning, and deep learning (DL; Figure 1). Specifically, supervised learning includes artificial neural network (ANN), support vector machine (SVM), decision tree, Random Forest (RF), naive Bayes classifier, and K-nearest neighbor (k-NN) algorithm. Unsupervised learning mainly includes clustering algorithms and association rule algorithms. DL contains convolutional neural networks (CNNs), recurrent neural networks (RNNs), and deep neural networks (DNNs) (13, 14). AI technology differs in its applications and limitations for different data types. Therefore, the accurate diagnosis of coronary artery disease can only be achieved by finding an appropriate intelligent mathematical model to match the CCTA imaging data.

## Status of AI Applications at CCTA

### AI-Optimized CCTA Imaging Technology

#### Reduce the Radiation Dose of CCTA Examination

Coronary CT angiography (CCTA) has high sensitivity and specificity for detecting CAD, but for a patient who needs long-term follow-up, multiple CCTA examinations would inevitably lead to the accumulation of radiation doses and increase the probability of radiation injury. Researchers have been developing various technologies to reduce the dose level for patients and to achieve low-dose CCTA examinations (15–17). If we only focused on reducing radiation dose while ignoring image quality, the accuracy of our diagnostics would be affected. AI technology can be used to lower radiation doses without affecting image quality in patients (Table 1). Wolterink et al. (18) suppressed the image noise caused by low-dose CT through the combination of CNN and adversarial CNN. Yang et al. (24) proposed a method of generative adversarial networks (GANs) for visual perception, which could reduce the noise level of the image in low-dose CT and preserve the key details of the images. Brodoefel et al. (25) reported that body size was an independent factor affecting the quality of CCTA images. To obtain the same image quality, the patients with large body sizes required higher tube voltage and tube current than patients with normal body sizes. Nevertheless, increasing the tube voltage and tube current would undoubtedly increase the patients' radiation dose (26). AI reduces the radiation dose by learning from CT images in regular-dose phases to remove noise from low-dose phases while maintaining image details (19). In addition, several studies have used DL methods, the radiation dose of CCTA has been significantly reduced by using a low scanning voltage, and the degree of radiation dose reduction is 36%−55.65% (19–23).

**Table 1:** Application of artificial intelligence to reduce the radiation dose of CCTA.

**Study**	**Year**	**Networks**	**Algorithm**	**ED (mSv)**	**Degree of radiation dose reduction (%)**
Wolterink et al. (18)	2017	CNN	Discriminator CNN	0.2	NA
Kang et al. (19)	2018	GAN	Cycle-consistent adversarial denoising network	NA	NA
Benz et al. (20)	2022	CNN	DLIR	0.8	43
Liu et al. (21)	2020	GAN	GAN, Adversarial CNN combined with CNN	0.91	55.65
Li et al. (22)	2022	DNN	DLIR-H	0.75 ± 0.14	54.5
Sun et al. (23)	2022	DNN	DLIR	0.57 ± 0.31	36

*DNN, deep neural network; GAN, generative adversary networks; CNN, convolutional neural network; CCTA, coronary computed tomography angiography; DL, deep learning; ED, effective dose; DLIR, deep learning image reconstruction; DLIR-H, high-strength deep learning image reconstruction*.

#### Reduce Image Noise

By using DL-based image reconstruction (DLR) and iterative reconstruction (IR), Takatsugami compared the quality of CCTA images processed by DLR and IR and also measured the noise in the image of the ascending aorta, the left atrium, and the ventricular septum in all the images (27). A contrast-to-noise ratio (CNR) for the proximal coronary artery was calculated as well. The results indicate that the average image noise for DLR images is lower than that for IR images (18.5 ± 2.8 vs. 23.0 ± 4.6 HU, *P* < 0.01) and CNR increased significantly (*P* < 0.01). With the DL image reconstruction method, Dominik C. Benz reduced the image noise by about 43% in comparison with the IR method (28). In addition, Hong et al. (29) applied DL to the removal of image noise by using an improved U-Net-type CNN; the denoised image was finally obtained by predicting the low-dose noise that might occur in the original model and then subtracting the prediction noise from the original noise. Image clarity was measured by edge rise distance (ERD), and the quality of the images was subjectively rated by two physicians. The results showed that the average ERD of the denoised image was significantly less than that of the original image (0.98 ± 0.08 vs. 0.09±0.08, *P*<0.001). In terms of diagnostic accuracy, there was no significant difference between the paired comparison groups. The study confirmed that, combined with IR techniques, the DL method could significantly facilitate noise reduction performance and image quality (Table 2).

**Table 2:** Application of artificial intelligence in reducing image noise.

**Study**	**Year**	**Algorithm**	**Degree of image noise reduction**	**Image quality mean scores (AI group vs. contrast group)**	**Mean image noise (HU) (AI group vs. contrast group)**	**ERD mean (mm) (AI group vs. contrast group)**	**Degree of radiation dose reduction**
Tatsugam et al. (27)	2019	DCNN	20%	3.58 vs. 2.96	18.5 vs. 23.0	16.7 vs.18.5	36%
Benz et al. (28)	2020	DCNN	43%	4.2–4.6 vs. 1.8–2.2	30 vs. 53	NA	65%
Hong et al. (29)	2020	CNN (U-net)	>20%	3.65 vs. 2.45	52.64 vs. 67.22	0.9141 vs. 0.9589	NA

*DCNN, deep convolutional neural network; CNN, convolutional neural network; ERD, edge rise distance; NA, not applicable*.

#### Reduce Motion Artifact of the Images

The coronary artery continuously supplies blood to the heart through regular contraction and relaxation. In patients with arrhythmia and high heart rates, the motion speed exceeds the scanning and the acquisition speed of CT equipment, resulting in motion artifacts in CT images, which influences the diagnostic accuracy and the reliability of coronary CT images and interferes with the evaluation of coronary lesions (30). In the branches of the coronary arteries, the motion direction of the right coronary artery is perpendicular to the CT scanning plane, which is more prone to motion artifacts. CT equipment with better hardware and higher resolution could be replaced for improvement, but the cost is too high. Thus, it is necessary to explore another approach to improve the image quality. Lossau et al. (31) believed that the time resolution of the CCTA images was limited by the angular range required for the reconstruction and the rotation time of the system. They modeled coronary motion artifact CT data to generate the data needed for AI and then used the trained CNN to iterate to an alternative path of a motion vector field (MVF) and motion compensated filtered back projection (MC-FBP), which effectively suppressed the artifacts caused by the angle in the image. After incorporating AI technology, the motion artifacts caused by heartbeat in CCTA images were significantly reduced, and the time resolution of the device was also made up.

#### Segmenting Automatically Decreases Postprocessing Time

Coronary CT angiography (CCTA) can estimate the origin and variation of branch coronary artery, the location of stenosis site, and the degree of stenosis. However, the process is time-consuming and energy-consuming and requires experienced doctors to take part in the analysis (32, 33). The lack of radiologists caused a large number of images from the CCTA to not be processed in time. Multiple study centers reported that AI could be used to automatically recognize the images and could mark and measure the lesions in advance (34–37). A radiology doctor only needs to proofread the reports that are generated by AI, which drastically increases diagnostic efficiency and reduces the probability of misdiagnosis or missed diagnosis.

Coronary artery segmentation is an important content in image postprocessing and the data should be collected according to the relative stationary phase in the individual cardiac cycle as the optimal time window (38). An optimal cardiovascular structure model should be constructed so that the physicians could evaluate the anatomy of the coronary artery from multiple perspectives and analyze the lesions. Kong et al. (39) developed a fully convolutional network (FCN) with tree structure, which involved the architecture model of multiscale discriminant feature extraction and final prediction and a tree structure layer for constructing anatomical structure. The model was carried out on four large-scale three-dimensional CCTA datasets. The final experimental results illustrated that it was more accurate and efficient than other methods of coronary artery segmentation. In several comparative studies, the accuracy of AI in segmenting coronary vessels is close to the manual, but the speed is much faster than manual (Table 3) (40–42).

**Table 3:** Application of artificial intelligence for image segmentation in CCTA.

**Study**	**Year**	**Algorithm**	**Degree of the post-processing time reduction**	**Post-processing time of the contrast group**	**Post-processing time of test group**	**DSC**
Kolossváry et al. (35)	2019	Radiomics-based ML	NA	NA	NA	NA
Podgorsak et al. (36)	2020	CNN	NA	25min	40 ms	NA
Kong et al. (39)	2020	FCN (Tree-structured CNN)	55%	DenseVox: 58 s ConvGRU: 26 s	26 s	0.8537
Huang et al. (40)	2018	CNN (3D U-Net)	NA	NA	NA	0.8291
Han et al. (41)	2020	CNN	85%	15–20 min	2–3 min	NA
Wan et al. (42)	2018	Hessian matrix	94%	Lankton's: 2min Zhang's: 1.53 s Li's: 29.90 s	1.72 s	0.93

*AUC, area under the receiver operating characteristic curve; CNN, convolutional neural network; CCTA-AI, CCTA-artificial intelligence; DSC, dice similarity coefficient; D, Dimensions; FCN, fully connected network; NA, not applicable*.

### AI-Assisted Diagnosis of CCTA

#### Coronary Artery Calcium Score

Coronary artery calcium (CAC) is a manifestation of coronary atherosclerosis. The formation of CAC is an organic, complicated, and controllable process. The coronary artery calcium score (CACS) is usually detected and calculated by CCTA, which could predict the cardiac events of asymptomatic individuals (43). The CACS can guide lipid-lowering therapy and patients with CACS > 100 are most likely to benefit from lipid-lowering therapy, thereby reducing the incidence of atherosclerotic cardiovascular disease events (44).

**Figure 2:**
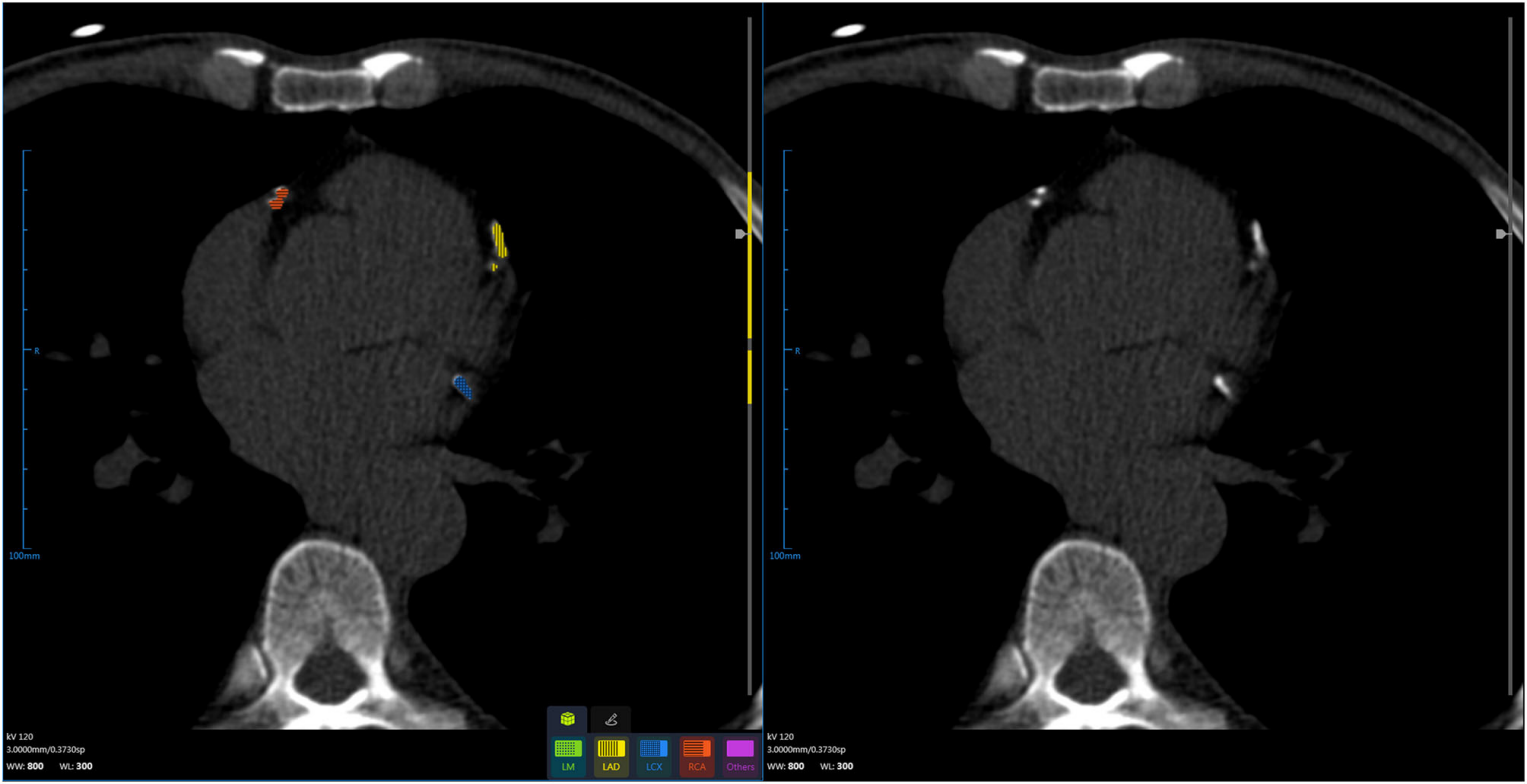
Using artificial intelligence, coronary artery calcification is identified, segmented, and scored.

The CACS calculation is a semi-automatic process that is required to draw the contour to obtain the region of interest (ROI) or click all the calcium-containing objects, which is usually time-consuming with manual intervention by the physicians (45). AI can accurately find and segment the vascular calcification through the algorithm and can automatically complete the calcification score, and then, the CACS was reviewed by the diagnostic physician; this prominently accelerates the process of the diagnostic system (Figure 2, Table 4). Wolterink et al. introduced a CNN algorithm, which could skip vessel segmentation and directly identify and quantify calcium. The results indicated that the CACS was very consistent with the reference quality score (46). June-Goo Lee demonstrated the high accuracy of AI on the CACS through a large sample study and used AI to perform risk stratification for CHD (47). D de Vos and van Assen (48, 49) used a CNN approach to accurately identify calcifications in cardiac and chest CT, extending automatic assessment of calcification scores to non-ECG-gated CT scans. AI can rapidly process CT images and calculate the CACS, which greatly alleviates the current shortage of medical talent. However, in reality, the CACS AI is still in its infancy, and it is only being piloted in a small number of hospitals. An important reason for this situation is the lack of large-scale clinical testing and validation of related AI software.

**Table 4:** Application of artificial intelligence in automatic coronary calcium scoring.

**Study**	**Year**	**Algorithm**	**ICC**	**κ**	**Accuracy**
Fischer et al. (45)	2020	RNN (LSTM)	NA	0.85	0.903
Wolterink et al. (46)	2016	CNN	0.944	0.83	83%
Lee et al. (47)	2021	CNN	0.99	0.94	NA
de Vos et al. (48)	2019	CNN	0.98	0.95	0.99
van Assen et al. (49)	2021	CNN	0.921	0.74	0.7

*CNN, convolutional neural network; CCTA, coronary computed tomography angiography; ICC, Intra-class correlation coefficient; κ, Cohen's linearly weighted kappa; NA, not applicable; CAC, coronary artery calcium; LSTM, long short-term memory; RNN, recurrent neural network*.

#### Analyze Coronary Plaque and Assess Risk

Atherosclerotic plaque and coronary artery stenosis are causally related. When plaque is accumulated on the coronary artery wall, it causes blood flow obstruction. When plaque continues to accumulate, it would lead to lumen stenosis and even myocardial ischemia, eventually resulting in myocardial infarction (50). In addition to the clinically explained qualitative characteristics of the disease, the volume of plaque is also correlated with the severity, progression, and prognosis of CHD (51).

**Figure 3:**
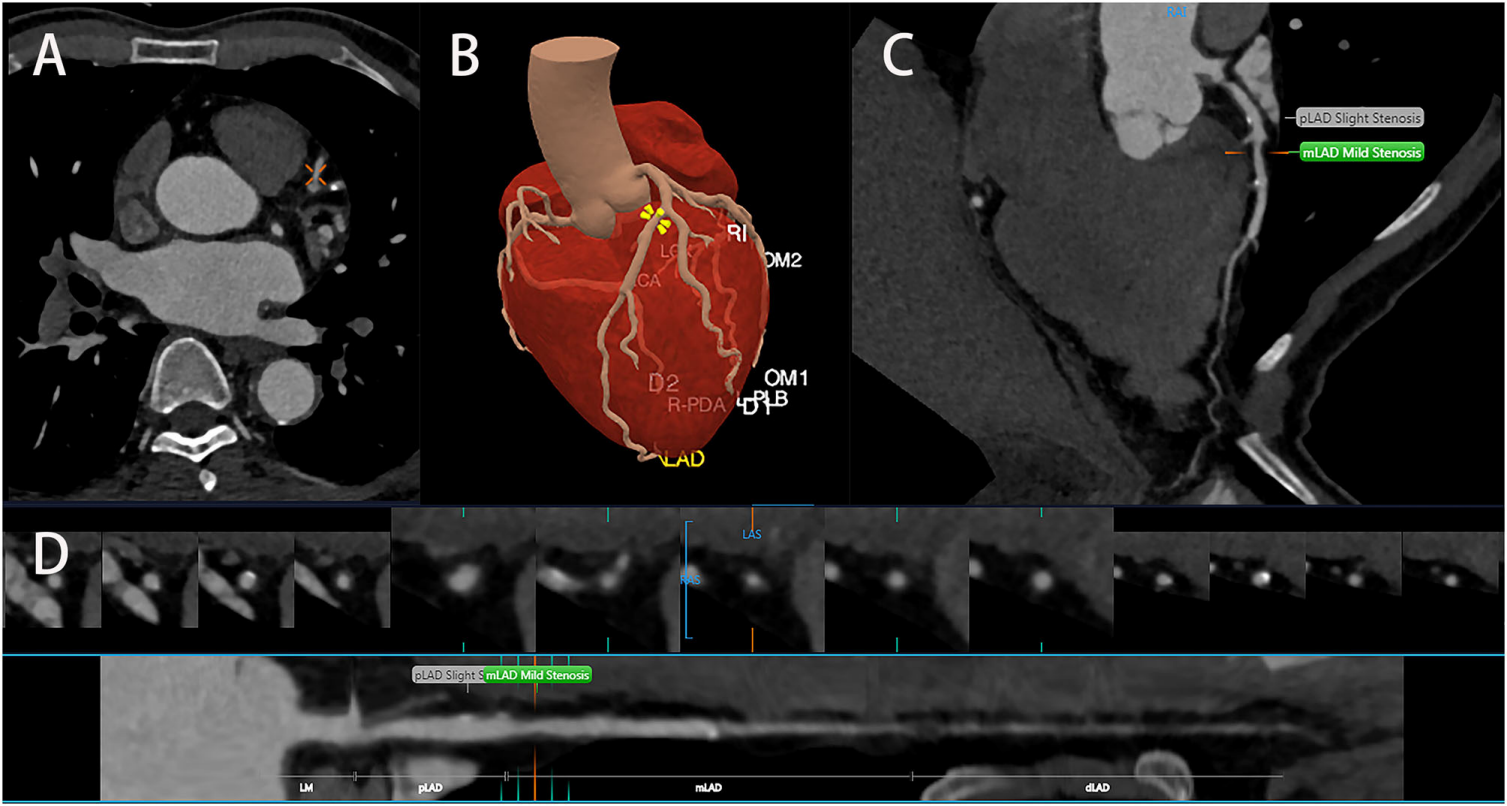
Artificial intelligence identifies coronary arteries **(A)** and segments them **(B)** accurately, identifies and classifies coronary plaques, and measures the severity of stenosis **(C,D)**.

Clinically, plaques can be classified into calcified plaques, noncalcified plaques, and mixed plaques. Plaques of different types would also cause different degrees of stenosis, and treatment methods will vary from person to person. The identification of noncalcified and mixed plaques is not as good as that of calcified plaques. In the face of a large number of CT images, missed diagnosis and misdiagnosis might be caused by visual fatigue. To detect patients who may suffer from CHD from the CCTA images, it is necessary to visually evaluate the plaque and measure the stenosis; this is a tedious and time-consuming process (52). AI can quantify the underlying concepts of textures and structures, input certain characteristics into the machine learning model, and automatically complete the plaque analysis and stenosis rate assessment, therefore greatly reducing the actual burden of imaging workers (Figure 3) (49). Majd Zreik et al. (53) used the CNN method to detect and classify the types of coronary plaques with an accuracy of 0.77; according to the CNN method, coronary plaque detection and classification by automated methods are feasible. The detection of vulnerable plaque is the importance of CCTA examination. The instability of vulnerable plaque increases the incidence of adverse cardiovascular events (54). Plaque could be affected by external forces, such as dynamic pressure or shear stress. Meanwhile, not all unstable plaques would contribute to cardiovascular events (55). AI is capable of extracting quantitative information through the algorithm integration of imaging data and it would also facilitate extracting vulnerable plaques more quickly and automatically and provide accurate decision-making based on multiple specific features of an anatomical segment. Kolosvalay et al. (35) introduced radiomic parameters into 8 machine learning algorithms. A total of 75% of the data were employed to train the ML model, and the remaining 25% of the data were visually evaluated and histogram evaluated using the feature area under the curve (AUC) and compared with the ML model. It was found that the ML model was better than visual evaluation in the identification of advanced atherosclerotic lesions. Tesche and Rosendael compared ML risk scores with conventional CT risk scores using the AUC, namely, the Agatston calcium score and the segment involvement score (SIS); they demonstrate that the ML model could improve the accuracy of risk stratification in plaque-derived information (56, 57). Their results indicated that the AUC of the ML model was significantly higher than that of the conventional CT risk score and that there was good agreement between the unstable plaque measurements and clinical parameters (including the Framingham Risk Score). ML could identify all the plaque information from CCTA and provide a more precise risk assessment. Gudigar et al. (50) selected 122 works of literature; they analyzed and summarized the methods and performance indexes of ML and DL, compared the artificial plaque classification scheme, and concluded the application of AI algorithms in plaque deposition prediction, detection, and classification. Statistics indicated that AI algorithms could provide valuable information for treatment decision-making and that ML- and DL-based AI algorithms were outstanding in identifying plaque.

#### Assess the Severity of Coronary Artery Stenosis

Coronary artery stenosis is a chronic result of long-term atherosclerosis (AS) accumulation caused by multiple factors and pathways. As a result of certain inflammation factors, coronary intima hyperplasias and carotid intima-media and adventitia thickened and later fibrosis was developed. In the end, coronary arteries can be stenotic or occluded (58). Therefore, early detection of coronary artery stenosis is important.

In recent years, AI technology was used to detect coronary artery stenosis, which can assist and improve diagnostic efficiency and accuracy (Figure 3D, Table 5). Chen et al. (59) applied a DL model to CCTA, took DSA as the diagnostic standard, and compared the detection performance of the DL model and the reader model at the level of each patient, each vessel, and each segment through the AUC. From the perspective of each patient, it could be seen that the diagnostic performance of the DL model (AUC = 0.78) was better than the reader model (AUC = 0.74) and that the diagnostic time (0.47min) was significantly less than the average diagnostic time of reader model (29.65 ± 2.15min). From the perspective of each vessel and segment, the diagnostic performance of the reader model was slightly better than that of the DL model. Kang et al. also proposed an ML algorithm different from Arnoldi, Kelm, and Goldenberg, which could detect obstructive lesions (stenosis rate ≥ 50%) and nonobstructive lesions (stenosis rate 25%−50%) (33, 60–62). In these examinations, they detected lesions of the left anterior descending artery, the left circumflex artery, and the right coronary artery in 42 patients, and the results discussed by three highly qualified specialist physicians were also compared. It was found that the algorithm performed well in the automatic detection of obstructive and nonobstructive lesions by CCTA, with a sensitivity of 93%, a specificity of 95%, and an accuracy of 94%. In conclusion, AI possesses high accuracy and efficiency in detecting coronary artery stenosis.

**Table 5:** The diagnostic performance of artificial intelligence in coronary stenosis.

**Study**	**Year**	**Methods**	**Sensitivity**	**specificity**	**PPV**	**NPV**	**Accuracy**
Kang et al. (33)	2015	SVM	93%	95%	NA	NA	94%
Chen et al. (59)	2020	DL	94%	63%	94%	59%	NA
Arnoldi et al. (60)	2010	Computer-aided	100%	65%	58%	100%	100%
Kelm et al. (61)	2011	Supervised Learning	97.62%	67.14%	NA	99.77%	NA
Goldenberg et al. (62)	2012	CAST	>90%	40%−70%	NA	> 95%	NA

*DL, deep learning; SVM, support vector machine; CAD, coronary artery disease; PPV, positive predictive value; NPV, negative predictive value; QCA, quantitative coronary angiography; CCTA, coronary CT angiography; CAST, computer-aided simple triage; NA, not applicable*.

#### CT-Derived Fractional Flow Reserve

The fractional flow reserve (FFR) is a tool conceived to assess the hemodynamic relevance of coronary plaques by measuring pressure differences across coronary stenosis (63). FFR functionally evaluates stenotic lesions. Nevertheless, the high cost and invasiveness of FFR are also the focus, which makes people more inclined to look for another inspection method. ML fractional flow reserve-CT (FFR-CT) is an emerging noninvasive functional examination for a combined anatomical and hemodynamic assessment of coronary lesions (Table 6). In the past, it used to take several hours to conduct computational fluid dynamics (CFD). In contrast, it took only a few minutes for the ML method, and the FFR-CT evaluation was in high consistency with traditional FFR examinations (68–70). Coenen et al. (64) took the FFR of coronary angiography as the reference standard; they recruited 351 patients and 525 vessels and compared the diagnostic performance of the FFR of CCTA by using the fluid dynamics method and ML methods. It was found that both the ML-based AUC (AUC=0.84) and CFD-based FFR-CT (AUC = 0.84) were better than the AUC of visual evaluation (AUC = 0.69, *P* < 0.001). The diagnostic accuracy of ML-based FFR-CT increased from 54%−63% to 75%−82%. In addition, Itu et al. (65) found that the evaluated FFR of the CCTA-based ML model was almost consistent with the CFD-based results, while the computational time of ML was reduced by 80 times. Tesche et al. (66) compared the technical performance of two methods to detect lesion-specific ischemia, namely, FFR derived from coronary CT angiography by computational fluid dynamics (FFR_CFD_) and FFR derived from coronary CT angiography by the machine learning algorithm (FFR_ML_). Each lesion and patient were sensitive to FFR_ML_ at 79 and 90% for detecting lesion-specific ischemia and specific at 94 and 95%. Based on each lesion and each patient, FFR_CFD_ produced a sensitivity of 79.0 and 89.0% and a specificity of 93.0 and 93.0%, respectively (*P* = 0.86 and 0.92). Compared with FFR_CFD_, FFR_ML_ had a significantly shorter processing time (40.5±6.3 vs. 43.4±7.1min; *P* = 0.042). Incorporating ML algorithms into CCTA not only improved the accuracy of diagnosis but also facilitated treatment decisions and outcome prediction. Coronary artery stenosis restricts the blood supply to the myocardium and might lead to ischemia and irreversible damage. The stenosis that significantly restricts blood flow should be treated invasively, whereas those that are minor should not be treated invasively (71).

**Table 6:** Application of artificial intelligence in CT-derived fractional flow reserve.

**Study**	**Year**	**Algorithm**	**Degree of acc progress (per-vessel basis)**	**Sensitivity**	**Specificity**	**R (ML, ICA)**	**PPV**	**NPV**	**AUC**
Coenen et al. (64)	2018	FFRML	78%	81%	76%	0.997	70%	85%	0.84
Itu et al. (65)	2016	FFRML	83%	82%	84%	0.729	69%	91%	0.9
Tesche et al. (66)	2018	FFRML	NA	79%	94%	0.81	87%	90%	0.89
Tesche et al. (67)	2020	FFRML	78%	82%	71%	0.63	70%	82%	0.84

*ICA, invasive coronary angiography; ML, Machine learning; FFR, derived fractional flow reserve; QCA, quantitative coronary angiography; NA, not applicable; PPV, positive predictive values; NPV, negative predictive values; AUC, area under the curve; FFRML, FFR derived from coronary; CT. angiography based on machine learning algorithm; R, Pearson correlation coefficient*.

However, there is a limitation in the ML algorithm. The quality of the image and coronary artery calcification would affect the diagnostic performance of ML-based FFR-CT. Coronary vessel segmentation is a crucial step in calculating FFR, and coronary calcification not only influences the accurate segmentation of vascular lumen but also overestimates the severity of vascular stenosis (72). Tesche et al. (67) investigated 314 patients (482 vessels in total) who first obtained the CACS, generated a patient-specific three-dimensional grid using the ML model, and calculated FFR-CT values throughout the coronary artery tree, using invasive FFR as a reference. It was reported that, with the increase in calcification score, the diagnostic accuracy of FFR-CT also decreased, but ML still had more prominent diagnostic advantages as compared to CCTA alone (69).

#### Epicardial Adipose Tissue and Perivascular Adipose Tissue

Pericardial fat contains pericardial adipose tissue (PAT), epicardial adipose tissue (EAT), and perivascular adipose tissue (PVAT). Among them, EAT and PVAT are close to the coronary artery and act directly on coronary atherosclerosis by the local release of inflammatory factors (73). Many studies have demonstrated that EAT and PVAT are independent predictors of adverse cardiovascular events (74–76). EAT deposits in the atrioventricular and ventricular sulcus, especially in the coronary subcutaneous vessel, and it is directly in contact with the coronary artery and its branches (77). The change in EAT thickness, therefore, may be associated with coronary artery disease in people with obesity (78, 79). In conclusion, the quantitative assessment of EAT contributes significantly to assessing coronary artery disease risk. However, quantitative analysis of EAT is obtained by manual measurement, which is very onerous. Commandeur et al. (80) proposed a fully automated quantitative tool to rapidly identify the pericardium and segment the epicardial and thoracic adipose tissues (TAT) from coronary calcium CT. Its results were more prominent compared to the improved version of the CNN with slice classification supervision (81). Commandeur combined two CNNs; they first segmented the heart and adipose tissues by a multitask CNN and then combined CNN and Statistical Shape Model (SSM) to detect the pericardium. The results showed that the agreement between expert and automatic quantification was good, with the median EAT volume of 78.03 cm^3^ [interquartile range (IQR): 57.08–105.79] and 78.64 cm^3^ (IQR: 54.48–106.58), respectively, and the correlation was 0.926 (*P* < 0.00001). This model is based on a deep CNN that improves the clinical guidance of EAT quantification for the diagnosis of CAD and improves the risk assessment for CAD. The PVAT can be considered as the adipose tissue around the blood vessels, and its attenuation changes can be measured by the fat attenuation index (FAI) (82). FAI reflects the differences in the peripheral coronary fat decay gradient and allows for direct visualization and quantification of coronary inflammation. When coronary artery inflammation occurs, PVAT changes its components to release proinflammatory cytokines and promote the hardening of the diseased vessel wall (83). Therefore, PVAT has an important clinical guiding value for CHD risk stratification and treatment (84). Antoniades et al. (85) proposed an AI-based image analysis method that captures perivascular attenuation gradients and reflects changes in PVAT biology caused by vascular inflammation; this method can improve the predictive ability of traditional risk stratification. Coronary artery inflammation can change due to the effects of drugs; FAI measurements are needed continuously to detect changes in perivascular adipose tissue composition, which is impossible (85). Crewe et al. (86) described fibrosis and vascular distribution based on specific texture patterns in PVAT radiomic profiles; this reflected the changes in the adipose tissues caused by chronic coronary inflammation and this algorithm significantly improved risk prediction of adverse clinical events. By combining FAI and FRP, Oikonomou et al. (83) detected adverse structural changes associated with PVAT fibrosis and microvascular remodeling. The results showed that FRP was not only significantly increased in patients with acute myocardial infarction (AMI) and that FRP remained unchanged 6 months after the event, while FAI decreased significantly after AMI. This indicates that FAI is a more dynamic measure of inflammatory biomarker and that FRP can capture more static changes. ML can also distinguish patients with acute myocardial infarction, patients with chronic CAD, or patients without CAD. Lin et al. (87) found that patients with acute myocardial infarction had significant peripheral adipose tissue radiological phenotype differences in patients with chronic coronary syndrome or without CHD; ML helps to identify patients with acute myocardial infarction.

Artificial intelligence can be more timely and can aid in the rapid analysis of the epicardial adipose tissue and the perivascular fat tissue with adverse fibrosis and distribution characteristics; AI can track the trend of coronary inflammation; AI also provides a more time-saving and more intelligent method for accurate assessment of dynamic CAD; this would help patients to reduce the incidence of adverse heart events (Table 7).

**Table 7:** Application of artificial intelligence in epicardial adipose tissue and perivascular adipose tissue.

**Study**	**Year**	**Methods**	**DSC**	**R**	**AUC (MACE prediction)**	**Median volume(cm^**3**^)(ML)**	**Median volume (cm^**3**^)(expert)**	**ICC**
Commandeur et al. (80)	2018	CNNs	0.823	0.926	NA	78.03	78.64	NA
Oikonomouet al. (83)	2019	Random forest, FRP	NA	NA	0.88	NA	NA	0.938
Lin et al. (87)	2020	CCTA-based radiomic analysis	NA	NA	0.87	88.9	NA	NA

*CNNs, convolutional neural networks; FRP, fat radiomic profile; DSC, Dice score coefficient; MI, myocardial infarction; PCAT, peri-coronary adipose tissue; AUC, area under the curve; R, correlation; NA, not applicable; MACE, major adverse cardiac events; AI, Artificial Intelligence; ICC, intra-class correlation coefficient*.

#### Prognostic Evaluation of AI on Coronary Artery Disease

Early detection and treatment of CAD are essential to avoid cardiovascular events. In recent years, CCTA, as the main means of a prognostic examination of CHD (8), has provided important prognostic information for CAD (88). Unfortunately, due to the differences in the clinical experience of diagnostic imaging physicians, there is a certain degree of subjectivity in the evaluation study and the characteristic information and details provided by CCTA would probably be missed. Consequently, the advantages of various AI algorithms are more obvious. AI can improve decision paths, risk stratification, and outcome prediction in a more objective, repeatable, and reasonable manner. These algorithms were learned from large training datasets and were then applied to task-specific prediction and intelligent decision-making of new untrained data (89). Patel et al. (90) evaluated the prognostic value of FFR-CT on myocardial ischemia using CCTA-derived parameters. The results indicated that, compared with patients possessing abnormal FFR-CT values, the patients with normal FFR-CT had a lower incidence of myocardial ischemia, less vascular remodeling, and a significantly lower risk of cardiovascular death or myocardial infarction.

Johnson et al. (91) collected CCTA data from 6,892 patients and then compared it with the Coronary Artery Disease Reporting and Data System (CAD-RADS) scores after evaluating the prognosis by ML methods. Based on all-cause mortality, the AUC of ML was 0.77, while that of the CAD-RADS was 0.72. Based on CHD mortality, the AUC of ML was 0.85, while that of the CAD-RADS was 0.79. The AUC of ROC of ML was higher than that of the CAD-RADS score. As an automatic analysis and diagnosis tool, AI can more acutely capture the prognostic information provided by CCTA and better improve the prognostic evaluation of CHD.

## Challenges and Prospects

As the main basis for disease diagnosis and treatment, huge amounts of data support the establishment of the medical image AI model. In the current status, the medical image AI system is still in the trial stage in cardiovascular diseases. The data provided for ML and modeling must be accurate, while it requires experienced doctors to label. As a result, relevant data resources are very scarce. In addition, there are deviations in the diagnostic standards of CAD in different medical institutions, and it is impossible to unify the quality and standards of data. Coupled with the issues such as data sharing and the lack of gold standards, the combination of AI and medical imaging has been hindered. In response to these problems, various medical research centers and relevant supervision departments should keep close contact to formulate data specifications and provide more important data support for the implementation of the AI automated auxiliary diagnosis system.

The realization of the combination of AI and medical imaging requires the sharing of data and the collection of these data needs the provision of basic personal information. Setting an encrypted entrance could not guarantee that the patient's privacy would not be disclosed as anyone might steal the patient's information and use it elsewhere. Therefore, the related functional departments are supposed to clarify the boundaries of medical ethics and pay close attention to the work of supervision; the personal information of the patients could be legally and compliantly managed.

Artificial intelligence has made a series of progress in CCTA image quality control, auxiliary diagnosis, and prognostic analysis, but it is still in the primary stage. The application of AI in CCTA will be more extensive and the diagnostic performance will be further facilitated. AI can further improve its value in auxiliary diagnosis, clinical prediction, and auxiliary decision-making, therefore achieving more accurate medical treatment, providing the patients with better individualized medical services, and promoting the development of cardiovascular medicine.

## Conclusion

Artificial intelligence has been used to automate the CCTA workflow, such as assessing coronary artery calcium, segmenting automatically, identifying plaques, and calculating the severity of stenosis. AI will play a greater role in the accurate assessment and prognosis analysis of CHD. However, before AI is widely used in clinical practice, there must be adequate measures done to ensure data security, data standardization, and so on.

## Author Contributions

JL and LH: conceived and designed the review and wrote the article. MQ: collected the data. BC: conceived and designed the review, contributed to the analysis of literature data, and wrote the article. GW: conceived and designed the review, contributed to the analysis of literature data, wrote the article, and acquired funding. All authors contributed to the article and approved the submitted version.

## Funding

This study was supported by the Guangdong Medical Science and Technology Research Foundation (A2021449).

## Conflict of Interest

The authors declare that the research was conducted in the absence of any commercial or financial relationships that could be construed as a potential conflict of interest.

## Publisher's Note

All claims expressed in this article are solely those of the authors and do not necessarily represent those of their affiliated organizations, or those of the publisher, the editors and the reviewers. Any product that may be evaluated in this article, or claim that may be made by its manufacturer, is not guaranteed or endorsed by the publisher.
